# Molecular and Cellular Effects of Hydrogen Peroxide on Human Lung Cancer Cells:* Potential Therapeutic Implications*

**DOI:** 10.1155/2016/1908164

**Published:** 2016-06-08

**Authors:** Gabriela Vilema-Enríquez, Aurora Arroyo, Marcelo Grijalva, Ricardo Israel Amador-Zafra, Javier Camacho

**Affiliations:** ^1^Departamento de Ciencias de la Vida, Universidad de las Fuerzas Armadas (ESPE), Avenida General Rumiñahui, S/N, P.O. Box 171-5-231B, Sangolquí, Ecuador; ^2^Centro de Nanociencia y Nanotecnología, Universidad de las Fuerzas Armadas (ESPE), Avenida General Rumiñahui, S/N, P.O. Box 171-5-231B, Sangolquí, Ecuador; ^3^Department of Physiology, Anatomy and Genetics, University of Oxford, Le Gros Clark Building, South Parks Road, Oxford OX1 3QX, UK; ^4^Department of Pharmacology, Centro de Investigación y de Estudios Avanzados del Instituto Politécnico Nacional, Avenida Instituto Politécnico Nacional 2508, 07360 Mexico City, DF, Mexico; ^5^Department of Genetics and Molecular Biology, Centro de Investigación y de Estudios Avanzados del Instituto Politécnico Nacional, Avenida Instituto Politécnico Nacional 2508, 07360 Mexico City, DF, Mexico

## Abstract

Lung cancer has a very high mortality-to-incidence ratio, representing one of the main causes of cancer mortality worldwide. Therefore, new treatment strategies are urgently needed. Several diseases including lung cancer have been associated with the action of reactive oxygen species (ROS) from which hydrogen peroxide (H_2_O_2_) is one of the most studied. Despite the fact that H_2_O_2_ may have opposite effects on cell proliferation depending on the concentration and cell type, it triggers several antiproliferative responses. H_2_O_2_ produces both nuclear and mitochondrial DNA lesions, increases the expression of cell adhesion molecules, and increases p53 activity and other transcription factors orchestrating cancer cell death. In addition, H_2_O_2_ facilitates the endocytosis of oligonucleotides, affects membrane proteins, induces calcium release, and decreases cancer cell migration and invasion. Furthermore, the MAPK pathway and the expression of genes related to inflammation including interleukins, TNF-*α*, and NF-*κ*B are also affected by H_2_O_2_. Herein, we will summarize the main effects of hydrogen peroxide on human lung cancer leading to suggesting it as a potential therapeutic tool to fight this disease. Because of the multimechanistic nature of this molecule, novel therapeutic approaches for lung cancer based on the use of H_2_O_2_ may help to decrease the mortality from this malignancy.

## 1. Introduction

Lung cancer is one of the main causes of cancer deaths worldwide [1]. Lung cancer can be divided into two major groups according to the pathological classification: small-cell lung cancer (SCLC) and non-small-cell lung cancer (NSCLC). NSCLC is a cancer of epithelial origin that comprises several histological subtypes that differ in their cytology, embryonic origin, anatomical location, and oncogene expression [2]. The most common subtypes of NSCLC are adenocarcinoma (40% of all forms of lung cancer), squamous cell carcinoma (25 to 30%), and large-cell carcinoma (10 to 15%) [3]. More than 80% of NSCLC display in many cases high metastatic potential and drug resistance, resulting in poor prognosis even with an early diagnosis [4]. Therefore, new treatment strategies are urgently needed.

Reactive oxygen species (ROS) are radicals, molecules, or ions with a sole unpaired electron in the outermost shell of electrons [5]. They are well known cytotoxic agents involved in the etiology of several human diseases including cancer. Accordingly, the expression of ROS detoxifying antioxidant proteins is altered in cancer cells in comparison to normal cells. For instance, thioredoxin reductase, thioredoxin, peroxiredoxin, glutathione S-transferase pi 1, glucose-6-phosphate dehydrogenase, and apurinic/apyrimidinic endonuclease 1/ref-1 (APE1/ref-1) have been found to be increased, while glutamate-cysteine ligase and *γ*-glutamyltransferase have been found to be decreased in lung cancer cells [6, 7]. APE1/ref-1 is a key enzyme in base excision repair and in the transcriptional modulation against oxidative stress. APE1/ref-1 is mainly localized in the nucleus of nontumor regions of the lung cancer tissue samples. However, nuclear and cytoplasmic expression of APE1/ref-1 is markedly upregulated in NSCLC, and the treatment of H460 lung cancer cells with hydrogen peroxide increases APE1/ref-1 expression [7].

ROS are considered potential carcinogens, since they play a role in mutagenesis, cancer promotion, and progression [8]. However, ROS also have anticancer properties by decreasing cell proliferation, damaging DNA, and inducing apoptosis, among other mechanisms. One of the most studied ROS is hydrogen peroxide (H_2_O_2_).

## 2. Hydrogen Peroxide

H_2_O_2_ is a protonated form of O_2_^2−^ and is produced in biological systems by the dismutation of superoxide anion in a reaction carried out by the enzyme superoxide dismutase (SOD) in the following manner [9]:

(1) Mn+1+-SOD+O2−⟶Mn+-SOD+O2Mn+-SOD+O2−+2H+⟶Mn+1+-SOD+H2O2,

where M = Cu(*n* = 1); Mn(*n* = 2); Fe(*n* = 2); Ni(*n* = 2).

H_2_O_2_ is also a soluble lipid and strong oxidizing agent that has been suggested to diffuse throughout the cell membrane via some aquaporins like aquaporin-8, AtTIP1;1, and AtTIP1;2 [10, 11]. H_2_O_2_ is also a hypochlorous acid precursor [9, 12]. This ROS reacts in the presence of transition metals like cupper or iron and produces the hydroxyl radical, a powerful reactive and toxic compound.

One of the preferred targets for H_2_O_2_ is the DNA; it produces single- or double-stranded DNA breaks as well as DNA cross links, in addition to purine, pyrimidine, or deoxyribose modifications [13]. Changes in DNA are usually repaired by the cell, but when persistent DNA damage occurs, then replication errors, genomic instability, activation of oncogenes, and inactivation of tumor suppressor genes might emerge [14]. All of these processes have been associated with the development of a variety of cancers. However, increasing evidence shows that H_2_O_2_ has contrasting effects on cancer cell proliferation depending on its concentration; it generates several antiproliferative responses, induces apoptosis, and inhibits cancer cell migration and invasion.

## 3. Effects of H_**2**_O_**2**_ on Plasma Membrane and Calcium Mobilization

**Figure 1:**
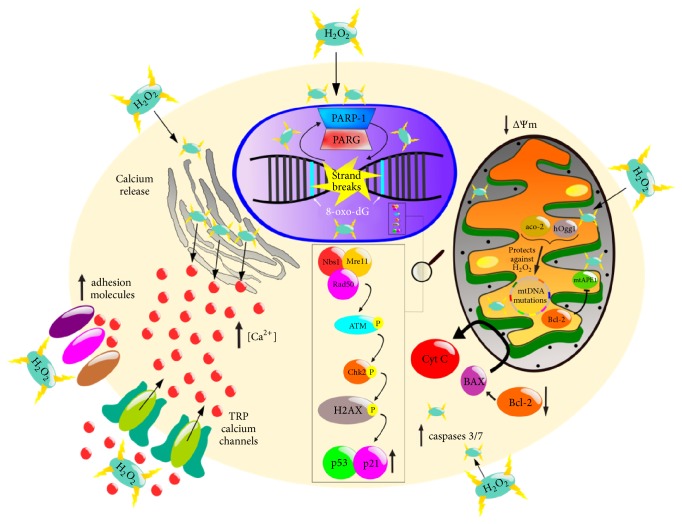
Effects of H_2_O_2_ that may lead to cell death. H_2_O_2_ affects several compartments and proteins potentially leading to cell death. Oxidation of DNA bases enriched with guanine adducts like oxo8dG base ring fragmentation, sugar modification, covalent cross linking of DNA and protein, and induction of DNA strand breaks may occur as a result of oxidative DNA damage induced by H_2_O_2_ [22]. Chk2 plays a major role in arresting the cell cycle progression in response to DNA damage [23]. Phosphorylation of Cdc25C and Cdc25A by Chk2 prevents cell cycle progression [24]. DDR involves the activation of the kinases ATM and Chk2 and their downstream effector p53 and its target p21^Cip1/Waf1^ axis [25, 26]. Overexpression of Aco-2 reduced oxidant-induced mtDNA lesions, mitochondrial p53 translocation, and apoptosis. Bcl-2 family proteins control the relocalization and actions of cytochrome C, a relevant step of apoptotic cell death [27]. H_2_O_2_ also increases caspase-3/caspase-7 activity [28] and upregulates the cleaved-caspase-9 [27], modifying the ΔΨm [28]. Apoptosis is also related with increased Ca^2+^ concentration that may be increased by influx via TRP ion channels or released from intracellular stores [29–31].

Ion channels play important roles in health and disease and Ca^2+^ signaling is an important second messenger that participates in many processes including proliferation and apoptosis. H_2_O_2_ increases intracellular Ca^2+^ concentration and decreases electrical resistance in human lung microvascular endothelial cells via activation of TRPV4 ion channels, through a mechanism that requires the Src tyrosine kinase Fyn [15]. In addition, exposure to H_2_O_2_ increases intracellular Ca^2+^ concentration in rat alveolar type II epithelial cells [16] and induces calcium release from the endoplasmic reticulum in endothelial cells [17]. Ma and collaborators observed that A549 lung cancer cells treated with H_2_O_2_ (500 *μ*M) showed an intracellular Ca^2+^ elevation due to Ca^2+^ influx and Ca^2+^ mobilization from intracellular stores. They also describe that H_2_O_2_ increases polyethylenimine/oligonucleotide endocytosis by activating the calcium/calmodulin-dependent protein kinase II (CaMKII). This study suggests that H_2_O_2_ may be useful to improving aerosol oligonucleotide therapy in pulmonary diseases [18]. Zhang et al. also observed that H_2_O_2_ increases the cytoplasmic Ca^2+^ concentration in A549 cells [19]; this change in calcium concentration might be a critical regulator of apoptosis. Another plasma membrane effect of H_2_O_2_ is on adhesion molecules that are important for permeability and signaling transduction in lung epithelium [20]. When A549 cells were stimulated by H_2_O_2_ the levels of the adhesion molecules CD49f, CD49b, CD29, and CD44 were increased. The expression of these molecules is closely associated with the stress response [21]. The effect of H_2_O_2_ on the plasma membrane and intracellular calcium concentration may be already involved in triggering cell death (Figure 1).

## 4. H_**2**_O_**2**_ Induces Nuclear and Mitochondrial DNA Damage

Cells are constantly exposed to reactive oxygen species including those metabolically generated as products of aerobic respiration [32, 33] and those originated from environmental pollutants [34]. It has been observed that hydrogen peroxide concentrations above 100 *μ*M are cytotoxic and genotoxic in A549 cells [35] causing DNA damage [36] and inducing the catalytic activities of DNA topoisomerase complexes [37–39]. Furthermore, the H_2_O_2_-induced damage could be also revealed by the oxidation of DNA bases, for instance, guanine adducts like 8-oxo-7,8-dihydro-2′-deoxyguanosine (8-oxo-dG) [40]. H_2_O_2_ DNA damage triggers a complex network of DNA damage response (DDR) pathways that may initiate DNA repair, arrest cell cycle progression, and trigger apoptosis. In A549 cells, H_2_O_2_ activates DDR through the Mre11 (MRN) complex of proteins (Mre11, Rad50, and Nbs1), which are essential for activation of telangiectasia mutated protein kinase (ATM), checkpoint kinase 2 (Chk2), and H2AX (*γ*H2AX). After Chk2 activation, the cells become arrested at either the G2-M or G1-S transition [41]. Moreover, total p53 and p21^Cip1/Waf1^ levels were increased after exposure of A549 cells to H_2_O_2_ [42]. These DNA damage response events induce the formation of DNA damage foci that probably will be activated by stalled replication forks, as well as by the induction of DNA double-strand breaks (DSBs) at the primary DNA lesion sites [43]. It has been described that H_2_O_2_ activates poly(ADP-ribose) polymerase (PARP) enzymes when DNA strand breaks have been paired, with the activation of PARP-1 and poly(ADP-ribose) glycohydrolase (PARG), suggesting that this activation process is a survival mechanism. Three members of the 17-member PARP family (PARP-1 to PARP-3) have been shown to be activated by DNA damage. Activated PARP enzymes cleave NAD^+^ into nicotinamide and ADP-ribose from which protein-bound (ADP-ribose)n polymers are synthesized; these polymers label the site of DNA damage enhancing DNA repair and consequently cell survival [44, 45]. Even though PARP activation has a central role in DNA single-strand break repair, its overactivation can cause cell death if excessive oxidative stress exists (in which DNA damage is severe and irreversible) [44]. On the other hand, repair of some DSBs can be error-prone resulting in deletion of base pairs and other defects that can result in translocations and chromosomal instability [46–49]. The association between DNA oxidation and DNA methylation in A549 cells exposed to H_2_O_2_ has been reported by Ke et al. Hydrogen peroxide induced the formation of 5-methylcytosine (5-mC), which is a cytosine variant produced by the transfer of a methyl group to the carbon located in the fifth position of cytosine. These authors showed that H_2_O_2_ induced decreased levels of DNA methylation in a dose-dependent manner, although significant changes in the level of DNA methylation required at least 10 days of exposure to the oxidant. This negative correlation suggests that DNA oxidation may take place before DNA methylation [40].

Not only nuclear DNA (nDNA) but also mitochondrial DNA (mtDNA) can be damaged by hydrogen peroxide. The rate of mtDNA mutations may actually be more than two orders of magnitude higher than that of nDNA. Somatic mutations of mtDNA are potentially more harmful for cell physiology compared to somatic damage of nDNA; consequently, the DNA repair systems may play a more important role in the mitochondria than in the nuclei, especially in nondividing cells [50]. This could be explained because mtDNA is in close proximity to the electron transport chain and due to the lack of protective histones [51, 52]. Even though the mechanisms that modulate mtDNA damage are still unclear, Kim et al. suggest that human 8-oxoguanine DNA glycosylase (hOgg1) and aconitase-2 (aco-2) are important factors in limiting oxidant-induced mitochondrial DNA damage. Thus, H_2_O_2_ induces nuclear and mitochondrial DNA damage by several mechanisms [53]. Human APE1 is a major component of the base excision repair in both nDNA and mtDNA [54] in various types of cells, including lung cancer cells [7, 55]. It has been shown that Bcl-2 suppresses mtDNA repair through direct interaction with APE1 in mitochondria via its BH domains and inhibition of mtAPE1 endonuclease activity [56]. This led to increased frequency of mtDNA mutations following H_2_O_2_ or nitrosamine 4-(methylnitrosamino)-1-(3-pyridyl)-1-butanone (a carcinogen in cigarette smoke) exposure in H1299 human lung cells [56]. Moreover, increased mitochondrial DNA (mtDNA) lesions in A549 cells have been reported after exposure to H_2_O_2_ in a dose-dependent manner; the effect also included a slight reduction in mtDNA copy number [53]. Figure 1 summarizes the effects of H_2_O_2_ on DNA damage.

## 5. Paradoxical Effects of H_**2**_O_**2**_ on Cancer Cell Proliferation and Migration

ROS have been proposed to have contrasting effects on cancer models. On one hand, ROS may promote cancer initiation; however, they can also inhibit metastasis of melanoma cells [57, 58]. Opposite or dual effects for H_2_O_2_ on cancer cell proliferation have also been described. For instance, H_2_O_2_ (50–200 *μ*M) inhibits the proliferation of human breast cancer MCF-7 cells [59], but at 1–10 *μ*M it increases the proliferation of hepatoma 7721 cells [60]. Interestingly, the proliferation of HT-29 colon cancer cells is enhanced at 10 *μ*M whereas a higher concentration (1000 *μ*M) leads to apoptosis [61]. H_2_O_2_ (50 *μ*M) also produced cell cycle arrest in A549 lung cancer cells; this effect correlated with the downregulation of cyclins D1 and E [62]. Cell migration and invasion are very relevant in cancer progression and malignancy. Opposite effects of H_2_O_2_ on these phenomena have also been observed. The migration of H460 large lung cancer cells was inhibited by 100 *μ*M H_2_O_2_; the superoxide anion and hydrogen peroxide downregulated Cav-1 expression and inhibited cell migration and invasion, whereas the hydroxyl radical upregulated Cav-1 expression and promoted cell migration and invasion. The downregulating effect of superoxide anion and hydrogen peroxide on Cav-1 was mediated through a transcription-independent mechanism that involved protein degradation via the ubiquitin-proteasome pathway [63]. In H1299, non-small lung cancer cells, 100 *μ*M of H_2_O_2_ inhibited migration, upregulated Deleted in Liver Cancer 1 (DLC1) protein expression, and reduced the activity of RhoA [64]. Thus, H_2_O_2_ may be used as an inhibitor of cancer cell proliferation, migration, and invasion if used at particular concentrations and cancer cell types. The potential use of this ROS as an anticancer agent is also supported by its proapoptotic properties, as the following discussed.

## 6. Hydrogen Peroxide Leads to Cell Death/Apoptosis

DNA damage responses usually end up with the decrease of cell viability and activation of apoptosis pathways depending on the stimulus intensity. H_2_O_2_ induces cell death/apoptosis [65–67] and attenuates cell viability of A549 cells in a concentration- and time-dependent manner [68]. One of the first damage mechanisms induced by oxidative stress is carbonylation of lipids, proteins, and DNA as it has been observed in A549 lung cancer cells [69]. Moreover, H_2_O_2_ decreases intracellular ATP levels and stimulates caspase-3/caspase-7 activity [28] and upregulates the expression of cleaved-caspase-9 [27]. This nonradical ROS also affects the mitochondrial membrane potential, closely related to mitochondrial-mediated apoptosis [28]. Cui et al. showed that H_2_O_2_ downregulates the antiapoptotic protein Bcl-2, upregulates the proapoptotic protein BAX, and increases cytochrome C (Cyt C) release from the mitochondria (Figure 1) [27]. It is well known that translocation of BAX from the cytosol to the mitochondria plays a role in the release of mitochondrial proteins [70]. Prolonged dissipation of mitochondrial membrane potential (ΔΨm) might result from mitochondrial DNA damage. Therefore, the upregulation of BAX and the loss of the ΔΨm produced by H_2_O_2_ may be responsible for the effect of BAX in the mitochondrial release of Cyt C in A549 cells. Indeed, the apoptotic intrinsic pathway is activated by several mitochondrial proteins released into the cytosol, including Cyt C [71]. In addition to its effects on apoptosis, H_2_O_2_ (100 *μ*M, 6–24 hours) induced necrosis in A549 cells [72]. Thus, H_2_O_2_ leads to cell death in different manners. Additionally, this ROS has effects on inflammation, as reviewed in the next section.

## 7. Inflammation, ROS, and H_**2**_O_**2**_ in Lung Cancer

Chronic inflammation has been proposed to play a central role in cancer development. Cancer-related inflammation is associated with the proliferation and survival of malignant cells, angiogenesis, tumor metastasis, and tumor response to chemotherapeutic drugs and hormones [73]. Thus, inflammation is a potential target for lung cancer prevention and treatment. Inflammatory cells release a variety of cytokines, chemokines, cytotoxic mediators including ROS, metalloproteinases (MMPs), and membrane-perforating agents, and soluble mediators of cell death, such as TNF-*α* (Tumor Necrosis Factor-*α*), interleukins (IL), and interferons (IFNs) [74]. The tumor stroma of NSCLC is characterized by active angiogenesis and abundant inflammatory infiltrate, which is mainly composed of tumor-associated macrophages (TAM). It is also characterized by the presence of tumor infiltrating lymphocytes (TIL), including T, B, and natural killer (NK) cells, and tumor-associated neutrophils (TAN) [75, 76]. Several important molecules involved in the inflammatory response are regulated by or have been associated with ROS and H_2_O_2_.

### 7.1. TGF*β*

Transforming growth factor-*β* (TGF*β*) is an immunosuppressive cytokine [77] that has a pleiotropic role in tumor biology and is frequently overexpressed in many cancers, including NSCLC [78–80]. TGF*β* affects cell growth, proliferation, differentiation, and apoptosis [81]. High expression of TGF*β* is a poor survival predictor in NSCLC [79]. Treatment of human malignant mesothelioma cells (HMM) with H_2_O_2_ promoted the epithelial-mesenchymal transition, as indicated by increased expression levels of vimentin, SLUG, and TWIST1 and decreased E-cadherin. Expression of stemness genes such as OCT4, SOX2, and NANOG was also significantly increased in HMM cells treated with H_2_O_2_. These gene expression changes were mediated via activation of hypoxia inducible factor 1 alpha (HIF-1*α*) and TGF-*β*1 [82].

### 7.2. Interleukins

Interleukin-10 (IL-10) is a multifunctional cytokine with both immunosuppressive and antiangiogenic functions; thus, it has both tumor-promoting and tumor-inhibiting properties [83]. Increased serum and peritumoral IL-10 levels have been reported in several malignancies [84], including lung cancer [83], suggesting a role for IL-10 in the tumor escape from the immune response. High IL-10 expression and increased serum concentrations of IL-10 in NSCLC patients have been shown to correlate with reduced survival [83]. IL-10 serum levels are higher in patients with metastatic disease in contrast to patients with localized tumors [85]. IL-10 favors tumor malignancy by promoting T cell apoptosis and tumor cell survival [86]. In lung carcinomas, IL-10 inhibits tumor cell susceptibility to cytotoxic T-lymphocyte-mediated killing [87]. Transgenic mice overexpressing IL-10 developed larger tumors than control mice when injected with Lewis lung carcinoma cells, suggesting that the production of IL-10 prevents a full immune response against the tumor cells [88]. IL-6 is of particular interest because it is expressed in malignant epithelial cells, and their expression is associated with a poor prognosis in lung cancer patients [89]. This interleukin has been detected in primary squamous cell carcinomas, adenocarcinomas, and several tumor cell lines [90, 91]. In a study with lung cancer patients, increased serum levels of IL-6 were found in 39% of the patients, whereas it was not detected in the serum of healthy controls or in patients with benign lung diseases [90, 92]. Bihl and coworkers demonstrated that IL-6 may be required for the proper control of cell proliferation in a subset of NSCLC cell lines. Two cell subgroups were reported in this study: NSCLC IL-6-dependent and IL-6-independent cells; this finding may have interesting clinical implications [93]. Paradoxically, antitumor effects of IL-6 have been demonstrated* in vitro* and* in vivo*, as well as in human biopsies from NSCLC and breast cancer [90]. TNF-*α* induced IL-8 gene expression in H441 lung epithelial cells by activating the IL-8 promoter via recruitment of NF-*κ*B to a TNF-*α* response element [94]. Similar results were obtained with lung adenocarcinoma GLC-82 cells treated with H_2_O_2_ (0.5 mM) [95]. In addition, Hsu et al. described that A549 lung cancer cells treated with H_2_O_2_ showed reduced I-*κβ* expression with a concomitant increase in NF-*κ*B and IL-8 expression [21].

### 7.3. NF-*κ*B

NF-*κ*B is a positive mediator of cell growth and proliferation as well as a critical signaling molecule in H_2_O_2_-induced inflammation. NF-*κ*B increases the expression of several components involved in cell cycle progression including cyclins D and E. However, the contributions of NF-*κ*B to lung cancer development are complex, and the underlying mechanisms are not fully understood [96]. Tumor biopsies from lung cancer patients showed high levels of NF-*κ*B activation in both SCLC and NSCLC and were significantly associated with TNM (tumor size, node status, and metastasis) stages and poor prognosis [96]. Interestingly, inhibiting NF-*κ*B with either siRNA, IKK inhibitors, or IKK suppressors inhibited lung cancer cell survival and proliferation [96, 97]. H_2_O_2_ activates cytosolic phosphorylation of NF-*κ*B p65 and ERK1/2 and induces nuclear translocation of pNF-*κ*B p65 producing inflammatory damage in A549 lung cancer cells. The genes involved in this response of the NF-*κ*B and MAPK signaling pathways included IL-1*β*, IL-6, IL-8, TNF-*α*, MCP-1, IP-10, and MIP [98].

### 7.4. MMPs

MMPs are a family of proteolytic enzymes that are capable of degrading various components of the extracellular matrix [99]. They are involved in all stages of cancer progression, not only in the process of tumor invasion and metastasis [100], but also in the proliferation, adhesion, migration, differentiation, angiogenesis, senescence, autophagy, apoptosis, and evasion of the immune system [101, 102]. Several studies have reported that plasma and/or serum levels of MMP-9 and TIMP-1 are elevated in stage III/IV lung cancer patients, when compared with patients with nonmalignant lung diseases [103, 104]. Retrospective studies of NSCLC tissue found that MMP-7 expression was higher in squamous cell carcinomas than in adenocarcinomas and correlated with significantly lower overall survival in patients [105]. MMP-9 is not produced by resident cells in the normal lung, but bronchial epithelial cells, alveolar type II cells, fibroblasts, smooth muscle cells, and endothelial cells produce MMP-9 in response to diverse stimuli [106]. Leukocytes in the lung can also be a source of MMP-9. Macrophages, eosinophils, mast cells, lymphocytes, NK cells, and dendritic cells all are able to produce MMP-9 [106]. Lung cancer cells, both primary and metastatic, can express MMP-9 constitutively, which may correlate with metastatic potential [106–108].

The transcription factor Ets-1 was found to be associated with the progression of several human cancers including NSCL [109]. Ets-1 may upregulate MMP-9 expression triggered by TGF-beta1 and TPA via MAPK signaling [110]. H_2_O_2_ upregulates Ets-1 via an antioxidant response element in the promoter, suggesting its potential role in ROS-triggered tumor progression [111]. Interestingly, H_2_O_2_ induced MMP-2 and MMP-9 expression in the lung adenocarcinoma cell line GLC-82, as well as of several components activated by the innate immune response including MyD88, TRAF2, TRAF6, and TRADD [95]. The association of ROS with inflammation might be used to suggest combined treatments of H_2_O_2_ with anti-inflammatory drugs in cancer therapy.

## 8. Potential H_**2**_O_**2**_-Based Therapeutic Strategies and Implications

Herein we described that hydrogen peroxide has several effects on lung cancer cells including DNA damage, cell cycle arrest, apoptosis, migration, and inflammation. Because many of these mechanisms end up with cell death, cautious delivery of H_2_O_2_ may be used as a potential therapeutic tool to treat some disorders including lung cancer. Actually, opposite effects of H_2_O_2_ may be also used in favor of some conditions. For instance, H_2_O_2_ (30 *μ*M) induced the migration of A549 cells, showing that the exposure to low concentrations of hydrogen peroxide may benefit tissue repair during acute lung injury [112]. Furthermore, H_2_O_2_ has been used to enhance the adhesion of hematopoietic stem/progenitor cells when systemically administered in inflammatory bowel disorders [113].

A few years ago, a hydrogen peroxide-generating system emerged as an interesting anticancer alternative strategy to selectively kill cancer cells. As cancer cells generate high concentrations of ROS and are under increased intrinsic oxidative stress, they might be more vulnerable to further oxidative insults produced by ROS-generating agents [114]. In malignant cells, prooxidant changes induce a redox shift that turns the cancer cell proliferative machinery on, leading to functional impairment, cell cycle arrest, and finally cell death. Even if the direct administration of H_2_O_2_ to cancer patients is not an accepted therapeutic strategy, there is now convincing evidence that H_2_O_2_-generating systems might be an efficient way of killing cancer cells [115]. For instance, H_2_O_2_ can selectively induce apoptosis in cancer cells and mediate, at least in part, the activity of several anticancer drugs including paclitaxel, doxorubicin, cisplatin, casiopeínas, and arsenic trioxide since these drugs generate ROS as a potential mode of action, increasing the rate of cancer cell death [116]. Actually, H_2_O_2_ seems to play an important role in oxidative stress-induced cancer cell death [115, 117]. H_2_O_2_ produced in the mitochondria is able to induce cell cycle arrest and senescence, a combination that might suppress tumor growth when sublethal concentrations of ROS are generated in response to therapy [118]. Not only synthetic products but also natural compounds have been described as promising candidates to potentially increase ROS levels and attack a wide variety of cancer cells. For instance, the codrug Bet-CA (a chemical combination of dichloroacetate and betulinic acid) increases ROS production and significantly alters mitochondrial membrane potential gradient (ΔΨm), followed by the release of Cyt C which prompts cells to undergo mitochondria mediated apoptosis [119].

Recently, cold atmospheric or nonthermal plasma has been suggested as an alternative therapy for different types of cancers with promising results obtained* in vitro* [120, 121] as well as* in vivo* [122, 123]. Nonthermal plasma can be produced by ionizing neutral gas molecules/atoms, which leads to a highly reactive gas at room temperature. This gas contains excited molecules and reactive species, among its most important constituents [123]. The therapeutic effects of nonthermal plasma result from the generation of ROS, which lead to ΔΨm, mitochondrial ROS accumulation, changes in the cell cycle, expression of DNA damage markers like *γ*H2AX, and finally induction of apoptosis [120, 121, 123]. Nonthermal plasma decreases the intracellular ATP concentration and the viability of A549 cells. It also increases the number of apoptotic cells due to caspase activation. In addition, plasma alters the mitochondrial membrane potential, regulates the mRNA levels of BAX, BAX1, H2AX, and Bcl-2, and modifies phosphorylated ERK1/2/MAPK protein levels [28].

Panieri et al. demonstrated that NSCLC cells resistant to conventional anticancer treatment can be sensitized in the presence of either high levels of H_2_O_2_ (48 *μ*M) resulting in DNA damage and irreversible ATP depletion (caspase-independent) or lower H_2_O_2_ concentrations (6.5 *μ*M) which induces inhibition of glycolysis and abrogation of ATP restoring mechanisms. Thus, cancers not responding to conventional therapies may be evaluated for their response to different H_2_O_2_ concentrations. Despite the fact that H_2_O_2_ may activate the inflammatory response potentially leading to cancer, the combined use of H_2_O_2_ with anti-inflammatory drugs may preserve the anticancer effect of this ROS and overwhelm the potential inflammatory response improving the anticancer treatment.

Recently, several drugs indicated for other diseases have been shown to have antiproliferative properties and have been suggested as an alternative therapy for different malignancies including lung cancer [124]. Thus, the novel combination of H_2_O_2_ with such repositioned drugs represents a new research area in cancer therapy.

## 9. Conclusions

**Figure 2:**
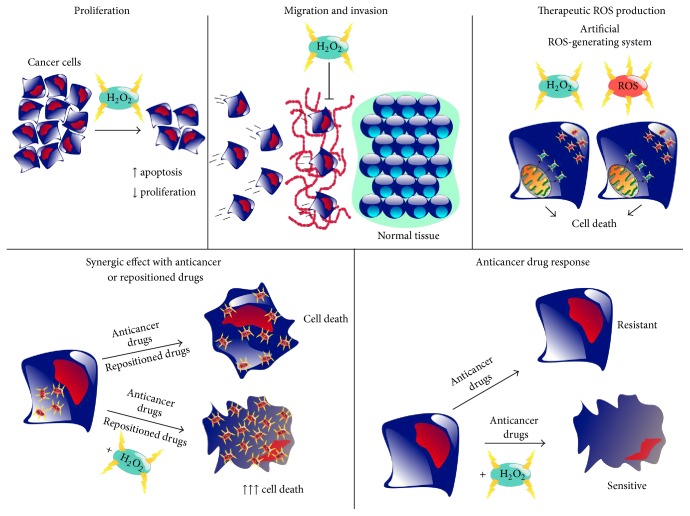
Potential therapeutic use of H_2_O_2_ to fight lung cancer. H_2_O_2_ can decrease the proliferation and increase the apoptosis of lung cancer cells. In addition, metastasis may be prevented because of the inhibitory effects of H_2_O_2_ in cell migration and invasion. Artificial ROS-H_2_O_2_ production directed to cancer cells in an excessive manner may lead also to cell death. H_2_O_2_ may also increase the cytotoxicity of anticancer drugs and revert drug resistance, as well as potentiating the effect of repositioned drugs with anticancer effects.

Because of the multimechanistic and multitarget anticancer properties of H_2_O_2_, this molecule is a very interesting potential therapeutic tool to fight cancer (Figure 2). The proper and cautious use of H_2_O_2_ in combination with commonly used chemotherapeutic drugs may have synergistic effects increasing lung cancer cell death. Particularly, novel therapeutic approaches combining H_2_O_2_ with repositioned drugs may help to decrease the mortality from this malignancy.

## Abbreviations

aco-2: Aconitase-2
APE1/ref-1: Apurinic/apyrimidinic endonuclease 1/ref-1
APE1: Purinic/apyrimidinic (AP) endonuclease 1
ATM: Activation of telangiectasia mutated protein kinase
ATP: Adenosine triphosphate
Bcl-2: B cell lymphoma 2
CaMKII: Calcium/calmodulin-dependent protein kinase II
Cav-1: Caveolin-1
CD49f: Cluster of differentiation
CDKs: Cyclin dependent kinases
Chk2: Checkpoint kinase 2
Cyt C: Cytochrome C
DDR: DNA damage response
DLC1: Deleted in Liver Cancer 1
DSBs: DNA double-strand breaks
ERK1/2: Extracellular-signal-regulated kinases
Fyn: Nonreceptor tyrosine kinase
H2AX: Histone family, member X
H_2_O_2_: Hydrogen peroxide
HIF-1*α*: Hypoxia inducible factor 1 alpha
HMM: Human malignant mesothelioma cells
hOgg1: Human 8-oxoguanine DNA glycosylase
IFNs: Interferons
IKK: IkB kinase
IL: Interleukins
IP-10: Interferon gamma-induced protein 10
MCP-1: Monocyte chemotactic protein 1
MIP: Macrophage Inflammatory Proteins
MMPs: Metalloproteinases
MRN: Complex of Mre11, Rad50, and Nbs1 proteins
mtDNA: Mitochondrial DNA
nDNA: Nuclear DNA
Nbs1: Nibrin
NF-*κ*B: Nuclear factor kappa-light-chain-enhancer of activated B cells
NK: Natural killer
NSCLC: Non-small-cell lung cancer
OCT4: Octamer-binding transcription factor 4
PARG: Poly(ADP-ribose) glycohydrolase
PARP: Poly(ADP-ribose) polymerase
Rad50: Double-strand break repair protein
ROS: Reactive oxygen species
SCLC: Small-cell lung cancer
siRNA: Small interfering RN
SOD: Superoxide dismutase
SOX2: Sex determining region Y-box 2
Src: Nonreceptor tyrosine kinase
TAM: Tumor-associated macrophages
TAN: Tumor-associated neutrophils
TGF*β*: Transforming growth factor-*β*
TIL: Tumor infiltrating lymphocytes
TIMP-1: Tissue metallopeptidase inhibitor
TNF-*α*: Tumor Necrosis Factor-*α*
TRPV4: Transient receptor potential cation channel subfamily V member 4
ΔΨm: Mitochondrial membrane potential.
